# Vital Sign Monitoring and Cardiac Triggering at 1.5 Tesla: A Practical Solution by an MR-Ballistocardiography Fiber-Optic Sensor

**DOI:** 10.3390/s19030470

**Published:** 2019-01-24

**Authors:** Jan Nedoma, Marcel Fajkus, Radek Martinek, Homer Nazeran

**Affiliations:** 1 Department of Telecommunications, Faculty of Electrical Engineering and Computer Science, VSB-Technical University of Ostrava, 17 Listopadu 15, 70833 Ostrava, Czech Republic; marcel.fajkus@vsb.cz; 2 Department of Cybernetics and Biomedical Engineering, Faculty of Electrical Engineering and Computer Science, VSB-Technical University of Ostrava, 17 Listopadu 15, 70833 Ostrava, Czech Republic; radek.martinek@vsb.cz; 3 Department of Metallurgical, Materials and Biomedical Engineering, University of Texas El Paso, 500 W University Ave, El Paso, TX 79968, USA; hnazeran@utep.edu

**Keywords:** fiber bragg grating (FBG), fiberglass, heart rate (HR), respiratory rate (RR), MRI-compatible, cardiac triggering, ballistocardiography (BCG)

## Abstract

This article presents a solution for continuous monitoring of both respiratory rate (RR) and heart rate (HR) inside Magnetic Resonance Imaging (MRI) environments by a novel ballistocardiography (BCG) fiber-optic sensor. We designed and created a sensor based on the Fiber Bragg Grating (FBG) probe encapsulated inside fiberglass (fiberglass is a composite material made up of glass fiber, fabric, and cured synthetic resin). Due to this, the encapsulation sensor is characterized by very small dimensions (30 × 10 × 0.8 mm) and low weight (2 g). We present original results of real MRI measurements (conventionally most used 1.5 T MR scanner) involving ten volunteers (six men and four women) by performing conventional electrocardiography (ECG) to measure the HR and using a Pneumatic Respiratory Transducer (PRT) for RR monitoring. The acquired sensor data were compared against real measurements using the objective Bland–Altman method, and the functionality of the sensor was validated (95.36% of the sensed values were within the ±1.96 SD range for the RR determination and 95.13% of the values were within the ±1.96 SD range for the HR determination) by this means. The accuracy of this sensor was further characterized by a relative error below 5% (4.64% for RR and 4.87% for HR measurements). The tests carried out in an MRI environment demonstrated that the presence of the FBG sensor in the MRI scanner does not affect the quality of this imaging modality. The results also confirmed the possibility of using the sensor for cardiac triggering at 1.5 T (for synchronization and gating of cardiovascular magnetic resonance) and for cardiac triggering when a Diffusion Weighted Imaging (DWI) is used.

## 1. Introductionn

In this article, we intend to introduce and describe a novel vital sign sensor developed at the VSB-Technical University of Ostrava and University of Texas El Paso based on the encapsulation of an FBG. Our primary motivation was to create a vital sign probe that would be small, lightweight and compatible with MRI and other electromagnetically dense environments. For encapsulation, we used fiberglass (fiberglass is a composite material made up of glass fiber, fabric, and cured synthetic resin). This type of encapsulation allowed us to create a sensor with minimal dimensions (30 × 10 × 0.8 mm) and low weight (2 g). Electromagnetic immunity (protection against interference from supply networks, electromagnetic field generating devices, etc.) is the main characteristic feature of our sensor. This characteristic allows us to monitor human vital signs in different environments affected by magnetic resonance, X-rays, and other electromagnetic fields. A detailed description of the encapsulation process and its effect on the fiber Bragg grating is described in Section 3.3.

During the research phase of this study, we focused on testing the functionality of the measuring probe. To achieve this, we first recruited ten volunteers (six women and four men), and after acquiring their written informed consents, we carried out preliminary vital sign measurements on them in a real MRI environment (1.5 T MR scanner). In clinical practice, it is critically important to explore the possibility of applying such sensors to monitor HR and RR in the harsh and claustrophobic MRI environments and be able to predict/detect hyperventilation states which may occur during such examinations [1,2]. Hyperventilation states occur when individuals begin to breathe quickly and heavily. These are accompanied by weakness, heart pounding and dizziness [2].

For prediction of hyperventilation, claustrophobic episodes and/or panic attacks special ECG devices with electrodes [3,4,5] are used (they are costly and have large dimensions and weights (tens of cm)). Thanks to the small size and light weight, our sensor minimizes the disturbance to the patient and offer a comfortable alternative to other types of vital sign sensors during both short- and long-term monitoring (except MRI environments for example in the sleep laboratories or in other long-term medical investigations).

**Figure 1:**
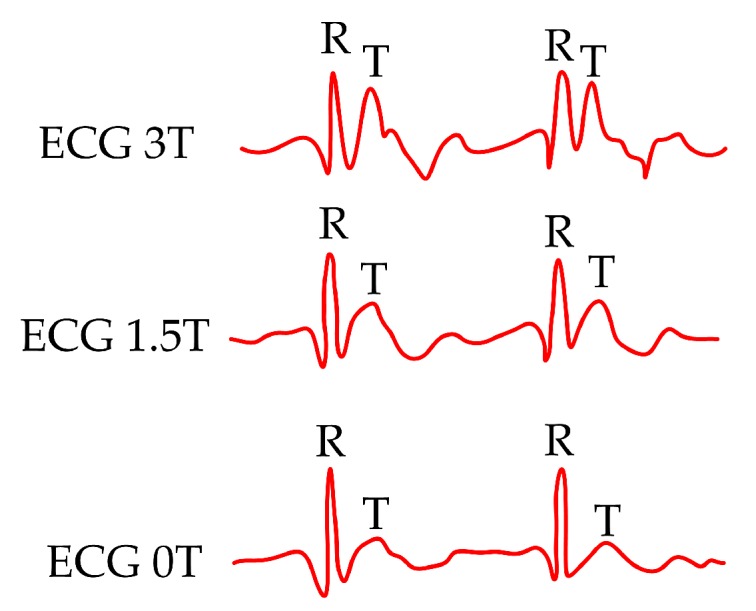
ECG signal in various types of magnetic fields (0 T, 1.5 T and 3 T).

Conventional ECG has a problem when applied in the MR environments. When a human body is placed in the magnetic field (magnetic field of MR scanner), an elevation of the T-wave of the ECG is frequently noted. This elevation may be so high that the T-wave becomes larger than the QRS-complex (also in some cases the R-wave may be reduced in amplitude) and is occasionally inverted please see Figure 1. Problems arise with the magnetic field at 1.5 T and grow with increasing magnetic field (3 T and 7 T), please see [6,7,8].

These above-mentioned effects may result in faulty cardiac triggering. Cardiac triggering is used to obtain static images at a particular time in the cardiac cycle. Triggering is a form of prospective gating where data (data acquired during MR examination) acquisition only begins after detection of a desired physiologic event (typically R-wave or peripheral pulse). Some type of ECG-based triggering is mandatory for imaging the heart of the human body [9] (and also is advisable for imaging of the lower part of the thorax). Also, cardiac timing measurements are clinically very important since cardiac timing exhibits millisecond precision, it is a good measure of myocardial cellular health, and irregularities in timing measurements. These facts are generally the first indication of problems in cardiac performance [10,11]. Problems of the influence of the magnetic field on the human body and heart activity can be solved by fiber-optic sensors. Using our sensor, the electrical activity of the heart was not sensed directly. Basically, our sensor sensed mechanical activity of the heart (more detail in Section 3.3 below). As discussed Brau et al. [12] fiber-optic technology (fiber-optic stethoscope) can be used for synchronization of Magnetic Resonance Imaging. We recognize another potential contribution of our work reported here because the sensor could also serve as an alternative substitute for the determination of the J wave. Basically, the J waves correspond to the R waves in the ECG signals which could be seriously disturbed during MR examinations. This is described in more detail in Section 3.2 below. This section also graphically and technically describes the R–J interval (R–J interval is defined as a time interval between R and J waves) that is important to define the use of our sensor within the cardiac triggering. Our presented results clearly confirmed, that measured signals is not affected by 1.5 T magnetic field (the sensor can be used for synchronization and gating of cardiovascular magnetic resonance, more in a discussion).

Another problem with existing ECG-based measurement in MRI environments is that the ECG signal may be degraded when the Diffusion Weighted Imaging (DWI) is used. The DWI—Diffusion Weighted Imaging—is a form of MR imaging based upon measuring the random Brownian motion of water molecules within a voxel of tissue. In simplified terms, highly cellular tissues or those with cellular swelling exhibit lower diffusion coefficients [13]. This results in a time lag with respect to the start of the given sequences in the individual measurement cycles (this in turn extends the duration of the examination, which leads to economic leakage due to the expensive MRI operation). We also recognize another potential contribution of our work reported here because the sensor could also serve as an alternative substitute for the determination of the T wave by detecting the J wave.

As our objective in this work was to address a clinical challenge, we first consulted with the Head Radiologist at the Faculty Hospital Ostrava (Assoc. Prof. Dr. Petr Krupa, M.D.), in the Czech Republic to guide and inform our sensor design efforts. Based upon his guidance and advice, we designed the novel sensor such that it’s shape would allow it to comfortably fit under a rectangular elastic belt. We then focused on the specifications of our sensor and verified its performance in a real MRI environment, all results were consulted with doctor Petr Krupa. Here, we describe the original results and accuracy of the HR and RR measurements performed by the tested sensor in comparison to corresponding reference measurements that are commonly used in clinical practice within MRI environments, also we discuss a possibility using our sensor for cardiac triggering (for synchronization and gating of cardiovascular magnetic resonance) and for the cardiac triggering when the Diffusion Weighted Imaging is used.

## 2. State-of-the-Art

The ECG records the bioelectrical changes that occur during the cardiac cycle—systole and diastole of the heart muscle. This is a recording of the differential changes in the bioelectrical voltages (biopotentials) that emerge during the evolution of the cardiac cycle as detected between certain anatomical sites on the skin with reference to ground (bioelectrode positions—defined as ECG leads). The ECG signal informs us about the position of the heart, the origin and transmission of the bioelectrical impulses and the Heart Rate (HR). The measurement of the electrical activity of the heart by the ECG is an established and well-recognized standard [14,15,16,17]. Currently, ECG modules using MRI-compatible bioelectrodes are fixed to a patient’s body to monitor their ECG signals. These modules are three-lead or 12-lead Electrocardiogram systems which are used for physiological monitoring of the patients during MR imaging, for example [18,19]. The acquired ECG information is wirelessly transmitted to a computer and processed by a software provided by the device manufacturer. Generally, these systems are characterized by high reliability and, of course, a high price tag.

There are several studies that describe the use of special devices called phonocardiograms for cardiac triggering. Brau et al. [12] discussed the fiber-optic stethoscope system for measuring cardiac activity that, unlike the ECG, is immune to electromagnetic effects. The optical system is shown to provide robust cardiac monitoring and gating signal in rats and mice during routine cardiac MR microscopy. Frauenrath et al. [20] demonstrated the applicability of acoustic cardiac triggering (ACT) for imaging of the heart at ultrahigh magnetic fields (7 T) by comparing phonocardiogram (based on a pressure transducer), conventional vector electrocardiogram (ECG) and traditional pulse oximetry.

Maderwald et al. [21] discussed an optoacoustic microphone for direct conversion of the acoustic to an optical signal at the subject and acquisition of the Phonocardiogram (PCG). Interesting results present Frauenrath et al. [22]. Authors discussed an acoustic gating device consists of three main components: an acoustic sensor, a signal processing unit, and a coupler unit to the MRI system. Signal conditioning and conversion are conducted outside the 0.5 mT line using dedicated electronic circuits.

Currently, Piezoelectric Respiration or Pneumatic Respiratory Transducers are mainly used to determine the Respiratory Rate (RR). They are applied, for example, in sleep laboratories [23] or MRI environments [24]. These are passive sensors (devices) that do not require electrical power to generate an electrical signal corresponding to the breathing period or respiratory rate. These sensors are fixed to an elastic band (belt) that is fastened around the chest. They generate a high-level, linear signal in response to changes in the thoracic circumference associated with respiration. These belts, however, cannot be used for quantitative respiratory volume measurements. The advantage, however, is that the respiratory rate detection belts can be very reliable, even with a variety of motion artifacts [25], providing a solid reference respiratory signal.

An alternative approach to monitor the RR and HR in magnetic fields is to use fiber-optics technologies. Fiber-optic sensors are gaining more popularity due to their flexibility, very small dimensions, reliability, independence from requiring an active power supply, and high immunity to electromagnetic interference (EMI).

Basic fiber-optic sensors include interferometric sensors using light interference. The use of these types of sensors for measuring Respiratory Rate (RR) is described in articles [26,27,28]. Michelson interferometers, where the sensor may be in direct or indirect contact with the body, are most commonly used to monitor Heart Rate (HR). The resulting measurement accuracy of these sensors is more than 95%, according to the authors in [29,30,31]. Mach–Zehnder interferometers [32], which are characterized by simpler and cheaper productions, have also been demonstrated. In addition, they can be made using tapers, as shown in publication [33], which is focused on monitoring the heart rate.

The use of Fiber Bragg Grating (FBG) to monitor RR or HR is becoming more popular, as evidenced, for example, by publications [34,35,36,37,38,39,40,41,42,43,44,45,46]. These publications describe, for example, various types of FBG encapsulations in materials such as polydimethylsiloxane (PDMS), which are characterized by their inertness to human skin and EMI. For example, Chethana et al. [36] reported a non-invasive optical ballistocardiography technique for the simultaneous measurements of cardiac and respiratory activities using an FBG Heart Beat Device. Their satisfactory results obtained from the EMI-proof sensor positioned around the pulmonic area on the chest were evaluated against an electronic stethoscope which detected and recorded the heart sounds. The mechanical package of their design used an intelligent combination of three simple components: A cone-shaped structure, a micrometer, and a flexible diaphragm. Their fabricated FBG sensor was tightly bonded to a diaphragm using a thin layer of cyanoacrylate adhesive. The study detailed in [43] describes the use of 2 FBG sensors encapsulated in PDMS polymers. In this work, the Bland–Altman (B–A) statistical analysis has shown accurate HR detection in multiple subjects. For the entire data set, 96.54% of the values fell within the ±1.96 SD range for HR determination. Textiles and bed mattresses can be also used to encapsulate FBG sensors to measure RR and HR simultaneously. Several techniques, such as weft and warp knitting, stitching, and weaving, can be used to embed the fiber sensing elements into textile fabrics [45,46].

Intensity variation-based sensors and polymer-based sensors discussed articles [47,48,49]. Chen et al. [47] demonstrated a highly sensitive micro-bend multimode fiber-optic sensor (accuracy of ±2 bpm for heart rate measurement) for simultaneous measurement of breathing rate and heart rate. The sensing system consists of a transceiver, micro-bend multimode fiber, and a computer. Krehel et al. [48] discussed flexible polymeric optical fibers integrated into a carrier fabric to form a wearable sensing system for respiratory rate monitoring. Authors of the article [49] present a polymer optical fiber sensor for simultaneous measurement of respiratory and heart rates. The sensor is characterized by errors below four beats per minute and two breaths per minute for the HR and RR and is embedded as a smart textile solution that can be used within the user’s clothes.

Practical measurements with fiber-optic sensors in real MRI environments, are described in publications [38,50,51,52,53,54,55,56,57,58,59]. The most interesting results have been presented in publications presented below. Dziuda et al. [38] reported results obtained from monitoring the respiration and cardiac activity of a patient during a magnetic resonance imaging (MRI) survey using an optical strain sensor based on an FBG. Their sensor was encapsulated inside a plexiglass to acquire ballistocardiographic signals from patients in MRI environments. For their entire dataset (three patients), these authors reported that 95.38% and 94.88% of the data were within the LoA (Limit of Agreement) range for the RR (0.85 rpm) and HR (3.29 bpm) determinations, respectively. The relative error level achieved in their study was below 8%. Article [51] describe three MRI compatible respiration sensors based on pure optical technologies. Smart medical textiles are now being developed that can sense elongation of up to 3% while maintaining the stretching properties of the textile substrates for patient’s comfort. Very small relative measurement errors were achieved by the authors of the study reported in [59], which focused on the use of an FBG sensor prototype (the sensor consisted of a fiber Bragg grating (FBG) attached to an elastic board) for measuring BCG signals not only in the sitting but also in the standing position of the body. Conventional ECG was used as the reference signal; the FBG sensor prototype was tested in the real MRI environment with a relative error of 1.8%. Interesting results are reported in [35], it is an overview article about fiber-optic techniques used for monitoring vital signs of the human body. A total of 47 MR-tested or potentially MR-compatible sensors have been described, but no sensor has been encapsulated in the fiberglass material. Table 1 lists the comparison properties of the most interesting prior MR studies of the fiber Bragg sensors designed for monitoring respiration and heart functions.

All of the above-mentioned FBG sensors used in real MRI environments use different constructions, encapsulations, and different ways of implementation on the human body or a variety of approaches in evaluating and processing the acquired information. In addition to introducing a small size and light weight sensor against previously published papers (Table 1), the main contribution of this article is attributed to the practical validation (original results) of an innovative FBG encapsulation method and testing of its functionality in a real 1.5 T MRI environment. The results also confirmed the possibility using sensor for the cardiac triggering at 1.5 T (more detail in Discussion). Another problem, as we mentioned above, is that the ECG signal may be degraded when the Diffusion Weighted Imaging (DWI) is used. Also, the authors see the potential of our sensor because the sensor directly not sensed the electrical activity of the heart, but the mechanical action of the heart (more in Section 3.3).

## 3. Methods

### 3.1. Fiber Bragg Grating (FBG)

**Figure 2:**
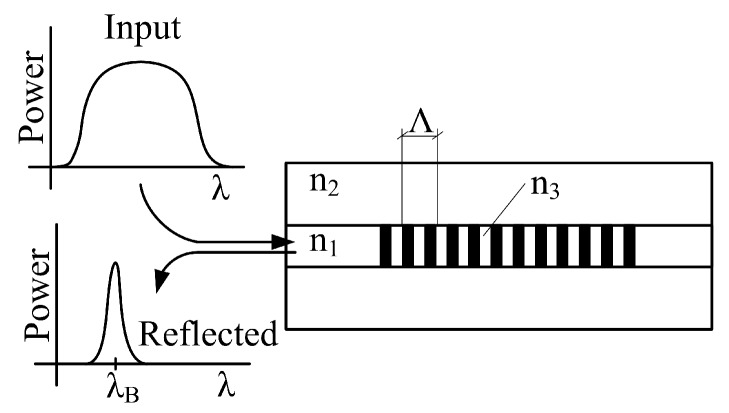
Blog diagram of the FBG structure.

An FBG is created by fabricating periodic changes in the refractive index of the core in an optical fiber, please see Figure 2. Designation $n_{1}$ represent refractive index of core, $n_{2}$ is the refractive index of cladding and $n_{3}$ is the higher refractive index of grating structure ($n_{3}=n_{1}+\delta_{n}$, where $\delta_{n}$ is the induced refractive index created during grating production). This periodic structure reflects a narrow spectral band of light, and the consequent wavelength shift is measured. This reflected wavelength is called the Bragg wavelength ($\lambda_{B}$). The central Bragg wavelength depends on the geometric and optical properties of the grating structure. The central Bragg wavelength can be expressed by the following equation:

(1) $$\lambda_{B}=2n_{eff}\Lambda ,$$

where $n_{eff}$ is the effective refractive index of the propagating mode and *L* is the period of refractive index changes.

Temperature and deformation depend on the central Bragg wavelength and other parameters. The temperature and deformation sensitivities are determined by normalized coefficients as follows. The FBG with the Bragg wavelength at 1500 nm shows the deformation sensitivity of 1.2 pm/μstrain and temperature sensitivity of 10.3 pm/${}^{\circ }$C. Encapsulation itself does not affect the deformation sensitivity, so for the our encapsulated sensor still applies the deformation sensitivity of 1.2 pm/μstrain [60]. The above-mentioned values of sensitivity apply to the Bragg grating created in the standard SMF-28 single-mode optical fiber (ITU-T recommendation G.652). This type of optical fiber was used for the production of FBG which we used. The normalized temperature coefficient is given by the following relation:

(2) $$\frac{1}{\lambda_{B}}\frac{\Delta \lambda_{B}}{\Delta T}=6.678\times 10^{-6}\;{}^{\circ }C^{-1};$$

and the normalized deformation coefficient is given as follows:

(3) $$\frac{1}{\lambda_{B}}\frac{\Delta \lambda_{B}}{\Delta \varepsilon }=0.78\times 10^{-6}\;\mu {\mathrm{strain}}^{-1},$$

where $\lambda_{B}$ represents the Bragg wavelength, $\Delta \lambda_{B}$ represents the shift of the Bragg wavelength, Δε stands for the change of deformation and ΔT symbolizes a change in the temperature [61].

### 3.2. Encapsulation Process of the Sensor

**Figure 3:**
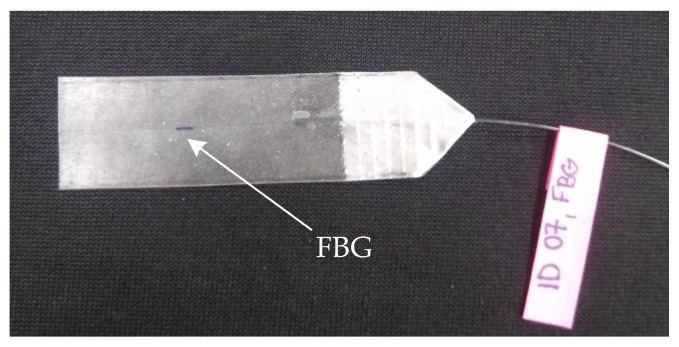
A prototype of created and designed measuring sensor.

For creating the measuring sensor, we used an apodised Bragg grating was made using the phase mask method at the producer Network group (Brno, Czech Republic). For the production process, we used a standard single-mode optical fiber (ITU-T recommendation G.652) with primary acrylic protection of 250 μm. This type of material and size of protection was used for the recounting at the place of the Bragg grating. The length of Bragg grating itself was 1.8 mm. For encapsulation grating structure we used a fiberglass (type Epikote Resin MGS LR 285 and Curing Agent MGS LH 285). Fiberglass is made of layers of glass fabric which are bonded by a resin and a curing agent (in our case a mixing ratio 2:1 was used). A total of 4 layers of glass fabric were used (2 layers—fiber Bragg grating—2 layers). Optical fiber with Bragg grating was oriented in the longitudinal direction of individual layers of the glass fabric. Curing time was 24 h at room temperature of 22 ${}^{\circ }$C. The measurement probe contains one connector of FC/APC type. Figure 3 shows a prototype of created and designed measuring sensor.

**Figure 4:**
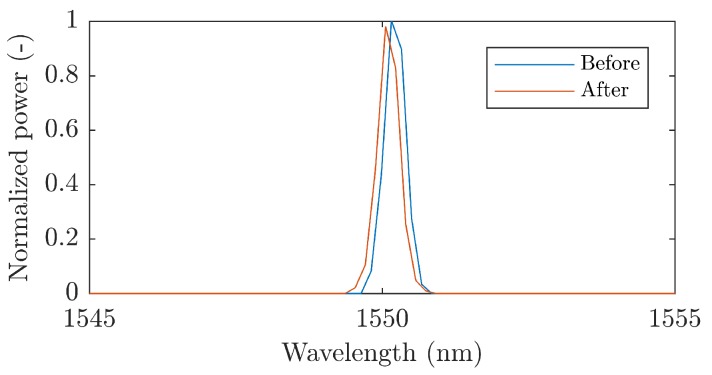
A reflection spectrum of the Bragg grating before and after encapsulation.

During a curing process, there is some shrinkage of individual layers of fiberglass. This has an influence on the geometrical dimensions of the Bragg grating structure. This change in geometrical properties leads to a change in optical properties and a shift of Bragg wavelength to lower wavelengths according to Equation (1). For encapsulation, we used a Bragg grating with a wavelength of 1550.218 nm. Due to the encapsulation process, the Bragg wavelength was reduced to the value of 1550.208 nm. Figure 4 shows the spectrum of the used Bragg grating before and after the encapsulation process. It can be seen that the encapsulation process has no significant effect on the shape of the reflection spectrum.

**Figure 5:**
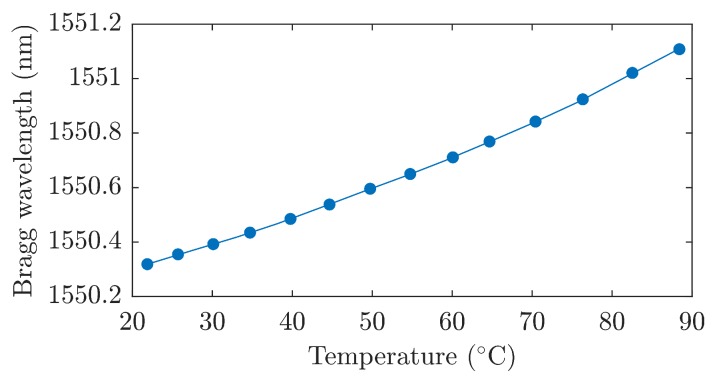
The dependence of Bragg wavelength on the temperature during the temperature loading in the temperature box.

Due to the cross sensitivity of the Bragg grating, temperature changes during mechanical stress measurements can cause measurement errors. In order to compensate for this error, it is necessary to know the temperature sensitivity of the sensor. The temperature sensitivity depends on the used Bragg grating encapsulation method. Determination of the temperature sensitivity was carried out by temperature loading of the sensor in the temperature box in the temperature range from 20 ${}^{\circ }$C to 90 ${}^{\circ }$C (the range of selected values is sufficient for bio applications). Figure 5 shows the dependence of Bragg wavelength on the temperature. It is evident that the temperature sensitivity increases from 10.3 pm/${}^{\circ }$C to 11.3 pm/${}^{\circ }$C due to encapsulation in the fiberglass. The presented results indicated that this type of encapsulation does not affect the structure and function of the FBG.

Because the sensor is in the form of a membrane, the own frequency of the membrane then can be defined as [60]:

(4) $$f_{hx,hy}=\frac{c}{2}\sqrt{{\left(\frac{h_{x}}{L_{x}}\right)}^{2}+{\left(\frac{h_{y}}{L_{y}}\right)}^{2}}$$

where $h_{x}$, $h_{y}$ are integer and *c* can be defined as:

(5) $$c=\sqrt{\frac{E}{\rho }},$$

where *E* is Young modulus, and ρ is the density of the membrane material.

Then we can write:

(6) $$f_{hx,hy}=\frac{1}{2}\sqrt{\frac{E}{\rho }}\sqrt{{\left(\frac{h_{x}}{L_{x}}\right)}^{2}+{\left(\frac{h_{y}}{L_{y}}\right)}^{2}}.$$

It is obvious that if the value of *E* is small, the membrane captures the low frequencies. Symbols $L_{x}$, $L_{y}$ represent dimensions of membrane.

On the contrary, if the value of *E* is bigger or big, the membrane captures high frequencies. Because fiberglass is characterised by the higher value of the Young modulus (type Epikote Resin MGS LR 285 and Curing Agent MGS LH 285), this material is suitable for capturing frequencies in the order of units up to tens of Hz (this range is so suitable for the biomedical applications). For the design of the sensor, the resonance frequencies lie in the order of hundreds of Hz to units of kHz. The shape and dimensions of the sensor are very important, which indicates the shape and range of the amplitude-frequency characteristic.

Due to the combination of the suitable Young modulus and shape and dimensions of our sensor, the sensitivity is high (as we present based on the Bland–Altman > 95%).

### 3.3. Grating Ballistocardiography

Our intention is to report on an FBG-based fiber-optic sensor used for measurement of RR and HR. Respiratory activity is physiologically manifested by the expansion and contraction of the chest. This breathing maneuver creates a periodic pressure change that acts on the FBG sensor positioned on the chest area (near the heart). Due to this pressure effect, the geometrical and optical properties of the FBG sensor change. In turn, these changes are manifested as spectral shifts in the reflected light, referred to as the Bragg wavelength, please see Equation (1) above. The consequent pressure modulation corresponds to the breathing activity. In the chest area, due to the high sensitivity of the FBG sensor, there is also a possibility to record a pressure effect caused by the cardiac activity (for our FBG sensor the response is typically 30–40 times weaker than the respiratory effect).

**Figure 6:**
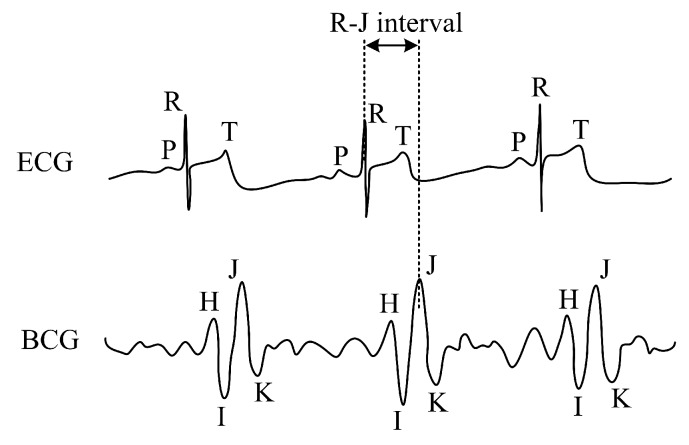
Sample recordings of electrocardiogram (ECG) and Ballistocardiography (BCG) signals.

The FBG sensor described here detects the mechanical action of the heart (the so-called Ballistocardiography—BCG signal). From a medical point of view, this is a more desirable form of non-invasive body movement sensing caused by the acceleration/deceleration of blood flowing inside large blood vessels. The flowing blood hits the aortic arch and this causes the body to move upwards and downwards [62]. Figure 6 below shows the comparison of ECG and BCG signals. The H wave is a concave wave beginning near the onset of the R wave and the I wave is a small wave following the H wave; the J wave, which is the largest wave of a concave shape following immediately after the I and the K waves, and is a convex wave.

The R–J interval (R–J interval is defined as a time interval between R and J waves) is important to define the use of our sensor within the cardiac triggering. The R–J interval was typically measured 250 ms for a healthy adult as shows paper [63], where authors used a static-charge-sensitive bed apparatus. In another study [64] was used weighing scale system and results are ranged from 203 to 290 ms for 92 healthy subjects participating in a study. For this article, similar results were found in [65] where authors presented an accelerometer-based BCG system. The R–J interval was measured around 133 ms.

### 3.4. Signal Processing

**Figure 7:**
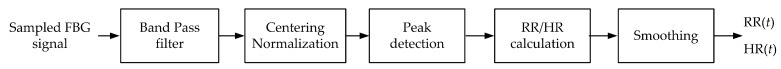
Signal processing from the FBG sensor to determine respiratory and heart rate [44].

To determine the respiratory and heart rate values, the signal from the FBG probe was processed according to the diagram shown in Figure 7. Unwanted signal components (such as motion artefacts, muscular activity, etc.) were produced by higher frequencies and these had to be filtered out. To determine respiratory rate, a third order Butterworth bandpass filter with cut-off frequencies from 0 to 0.5 Hz was used. Furthermore, normalization and centering of the signal to the zero mean value were performed. After this stage, the signal peaks were detected. Based on time marks, the Respiratory Rate (RR) was calculated according to the following equation: $RR=60/(t_{n}-t_{n-1})$, where $t_{n}$ was the time mark of the *n*-th peak and $t_{n-1}$ was the time mark of the preceding peak. The next step was to smooth out the respiratory rate curve. A median filter with a window size = 3, which was statistically more robust was used for this purpose [44,66].

A same bandpass filter with a cut-off frequency from 5 to 20 Hz was used to determine the HR. These values were calculated based on approaches detailed in [44,66,67]. As such the filtered signal provided a more reliable representation of the cardiac activity. The processed signal was then centered and normalized, and the peaks were detected. The heart rate (HR) was calculated by using the following relationship: $HR=60/(t_{n}-t_{n-1})$. A median filter with a window size = 7 was used for smoothing the HR values over time.

## 4. Experimental Setup

The experimental part is focused on the evaluation of the detected time series acquired during different experiments. The reported statistics is based on 3 h and 41 min of experimental data. After giving their informed written consents, 10 test subjects (volunteers) participated in this study. All test volunteers: 6 men (M1–M6) and 4 women (F1–F4) were tested in the supine body position. The MRI examination duration of the test subjects was different, please see Table 2 and Table 3. Also, we tried to test and analyzed the FBG sensor during different types of MR examinations (for certain types of MR examination, there is no need for interaction between the doctor and the patient, i.e., the patient is lying calmly throughout the examination. For other types of examination, the doctor asks the patient to cooperate—for example, by holding breathing during the examination, communication with a doctor, etc.).

**Figure 8:**
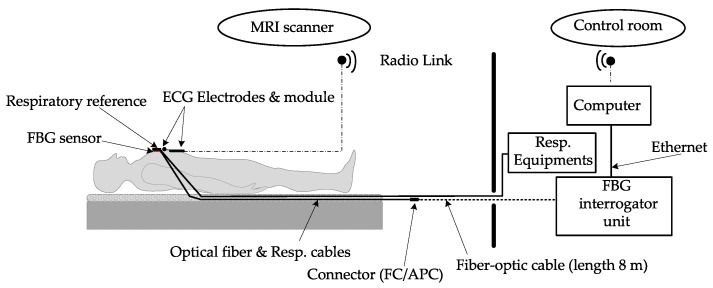
Positioning of the FBG sensor on the human body and the experimental setup.

The age group of volunteers was from 24 to 39 years and their weight was from 53 to 104 kg. A conventional three-lead electrocardiography (ECG) trigger device (Siemens Healthcare GmbH, Erlangen, Germany) was used as a reference for monitoring HR. This is a special MRI-compatible module and electrode system that is fixed to the monitored person’s chest, please see Figure 8. Wireless signaling was used in conjunction with a computer and the associated software provided by the device manufacturer; a signal was then filtered and processed.

**Figure 9:**
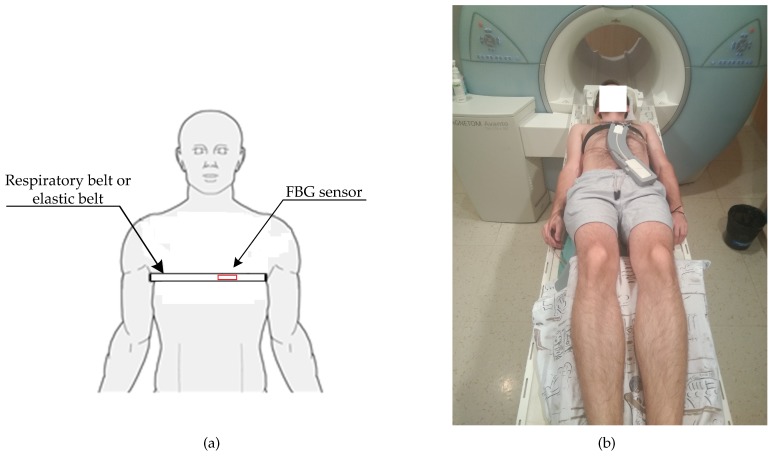
(**a**) a schematic location of the FBG sensor on the human body; (**b**) a photo taken during a real measurement (test subject M1).

As an optical interrogator unit, we used a spectral conventional instrument called the FBGuard [68]. The FBGuard is an FBG interrogation unit designed for measurement and processing of data measured by FBG sensors. The wavelength range of the used interrogator unit was between 1510–1590 nm, the wavelength resolution was ≤1 pm as provided a manufacturer. The FBGuard unit working with the output power of 1 mW. For our experimental tests, we used a sampling rate of 1 kHz, which appeared to be sufficient based on our previous research [44]. The position of our prototype FBG sensor on the human body and our measurement scheme is shown in Figure 8. The FBG sensor was placed around the pulmonic area near to the heart on the tests subject’s chest; please see Figure 9a. In our study, the sensor was positioned horizontally against the human body. The sensor was designed as part of a contact elastic belt that could be positioned on the patient’s chest (this elastic belt can be replaced by elastic belts with Pneumatic Respiratory Transducers or elastic belts used with Integrated Piezo-electric Respiration Transducers in the case of monitoring patients in sleep laboratories or long-term health clinics). The belt helps to securely attach the FBG sensor on the human body and holds it in place. The waist is tightened in such a way that it does not impose patient discomfort. Typically, it is tightened as when using breathing waist. We found that the effect of tightening on sensitivity is minimal (negligible). In our study, we used a Pneumatic Elastic Respiratory Transducer (type TSD221-MRI) as a reference for the measurement of RR (thoracic placement). The acquired respiratory signal was then processed according to instructions provided by the device manufacturer [69].

The experimental sensor data were compared with the reference measurements by using the objective Bland–Altman method [70]. The Bland–Altman analysis is a numerical and graphical method to compare two measurement techniques (in this case, sensor and reference data.) In this method, the differences between the data acquired by two different means are plotted against their average values. The reproducibility is considered to be good if 95% of the results lie within a ±1.96 SD (Standard Deviation) range.

Figure 9b shows a photo taken while performing a real measurement (test subject M1). A standard telecommunication optical cable (shown in dashed line in Figure 8) with a length of 8 m (SMF, ITU-T, G.652.D) was used for connecting FBG sensor and the interrogator unit (FBGuard). A Siemens Magnetom Avanto 1.5 T MR scanner was used in our experiments. During the examinations, no negative effects on the MRI function were recorded (the FBG sensor was not visible in the MR pictures, i.e., the recording was not compromised). This fact is further discussed in the discussion.

**Figure 10:**
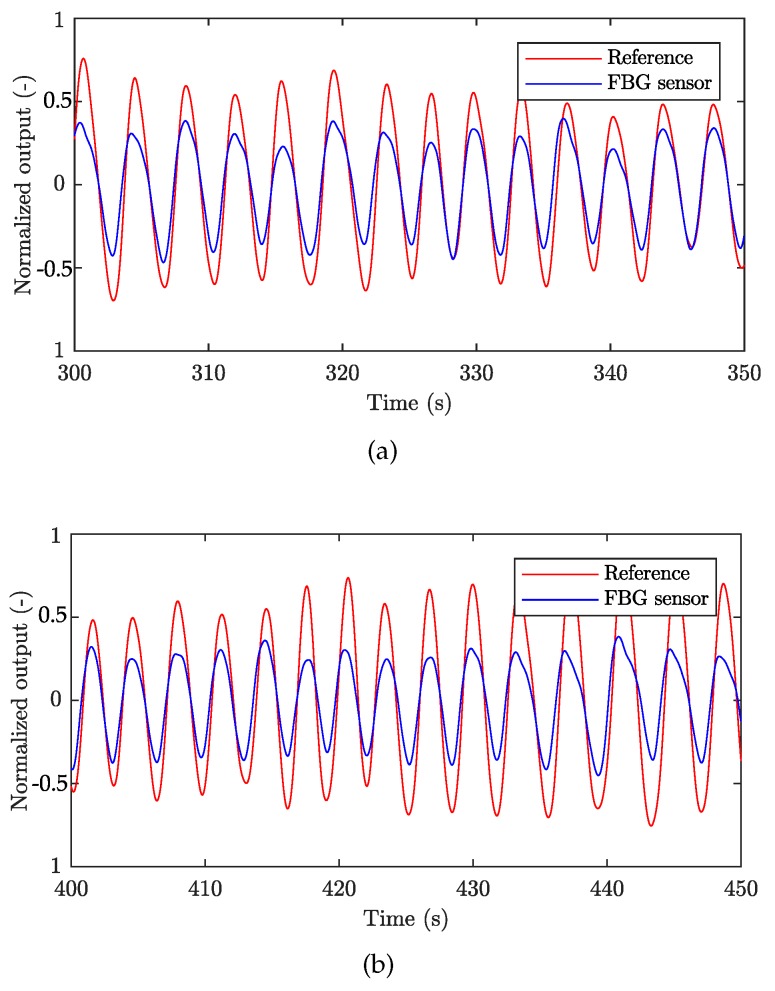
A sample recording of breathing activity using the FBG and the reference device: (**a**) M1 volunteer, and (**b**) F1 volunteer.

Figure 10a,b show a sample recording of the respiratory signal acquired from M1 (Figure 10a) and F1 (Figure 10b) volunteers, respectively. The signal represents a 50-second recording interval. The amplitude signals were normalized to adjust for unit differences on the vertical axis. The blue curve characterizes the signal obtained by the FBG sensor, and the red curve depicts the signal acquired from the Pneumatic Respiratory Transducer (reference).

**Figure 11:**
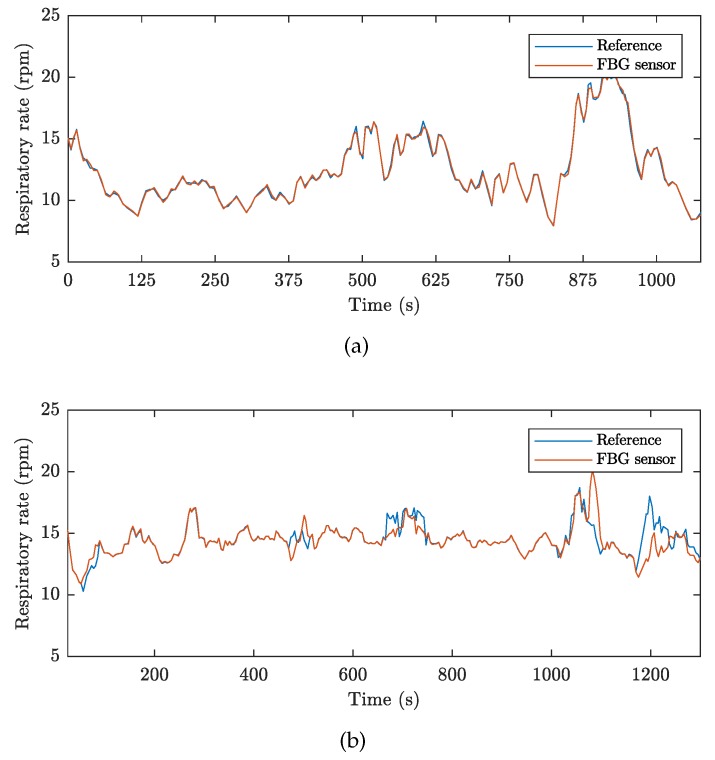
The full-time course of breathing activity over the conducted MRI examinations: (**a**) volunteer M1, and (**b**) volunteer F1.

For better clarity, the time course measurements of the breathing activity in volunteers M1 and F1 are shown for the duration of the conducted MRI examinations. Figure 11 represents a full-time course of respiratory activity (FBG sensor and reference) obtained from these 2 volunteers: M1 (17 min and 40 s) and F1 (21 min and 16 s). The RR is expressed in RPM (Respiration Per Minute). The graphs show that the signal obtained from the FBG sensor quite accurately reproduces the signal obtained from the reference device. This fact (including the statistical data from the remaining nine test volunteers) is described in Table 1 and is further confirmed by the Bland–Altman analysis.

**Figure 12:**
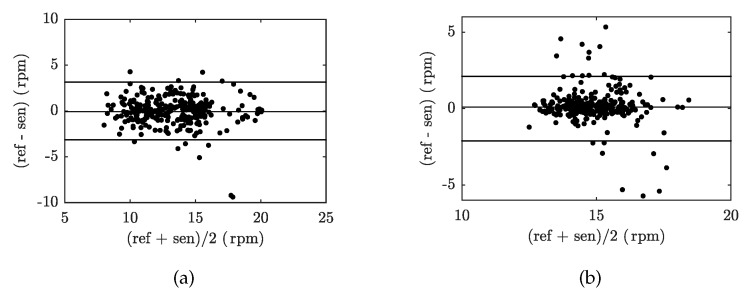
Reproducibility of RR determination capabilities of our FBG sensor (based on comparison with Pneumatic Respiratory Transducer-based RR calculations) using the Bland–Altman method for the data acquired from: (**a**) volunteer M1, and (**b**) volunteer F1.

Figure 12 shows the Bland–Altman graphs for the data acquired from the above-mentioned tested volunteers M1 and F1. The differences between the sensor and the reference traces, $x_{1}-x_{2}$, are plotted against their average values: $(x_{1}+x_{2})/2$.

**Figure 13:**
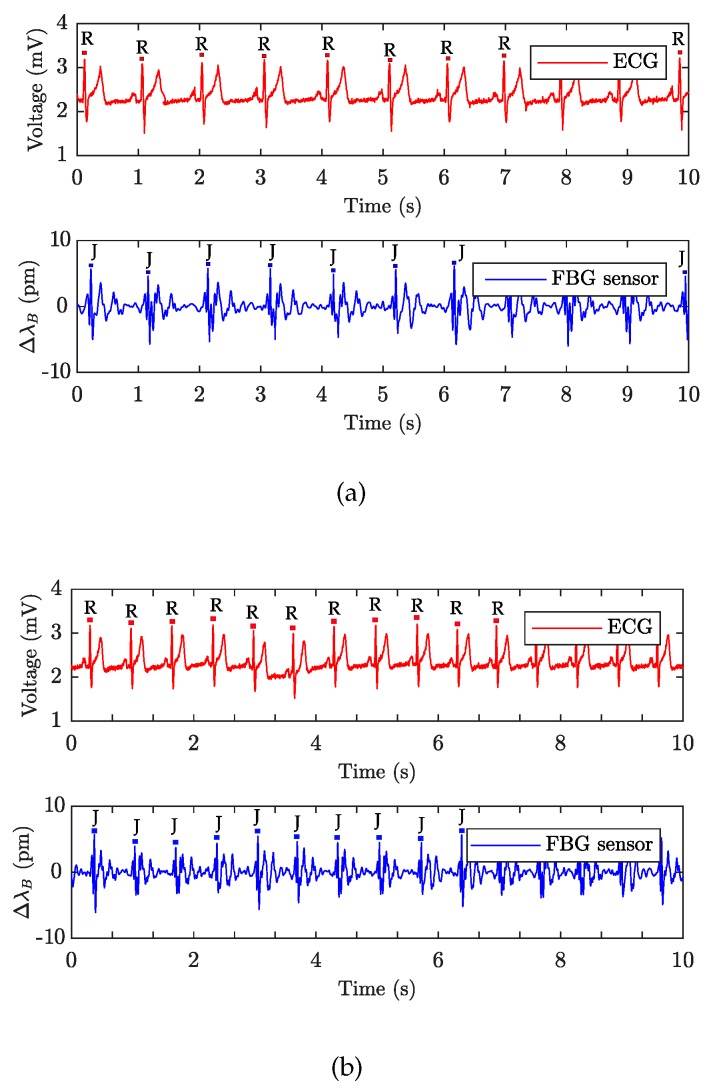
Ten-second long sample recordings of the heart activity; (**a**) M1 volunteer, and (**b**) F1 volunteer.

Figure 13a,b show sample recordings of the cardiac activity acquired from subjects M1 (Figure 13a) and F1 (Figure 13b), respectively. These recordings represent a 10-second recording interval. The blue curve characterizes the signal obtained by the FBG sensor, and the red curve depicts the reference ECG signal. In the case of the ECG signal, the *Y*-axis is given in millivolts (mV) and in the case of the FBG sensor it represents the shift of the Bragg wavelength ($\Delta \lambda_{B}$) expressed in (pm). The individual maxima detected in the ECG signals represent the R waves, and each maximum value (peak) detected in the FBG sensor recordings characterizes the J wave (as it was shown in Figure 3, the J wave in the PCG signal is a representative of the R wave in the ECG signal).

**Figure 14:**
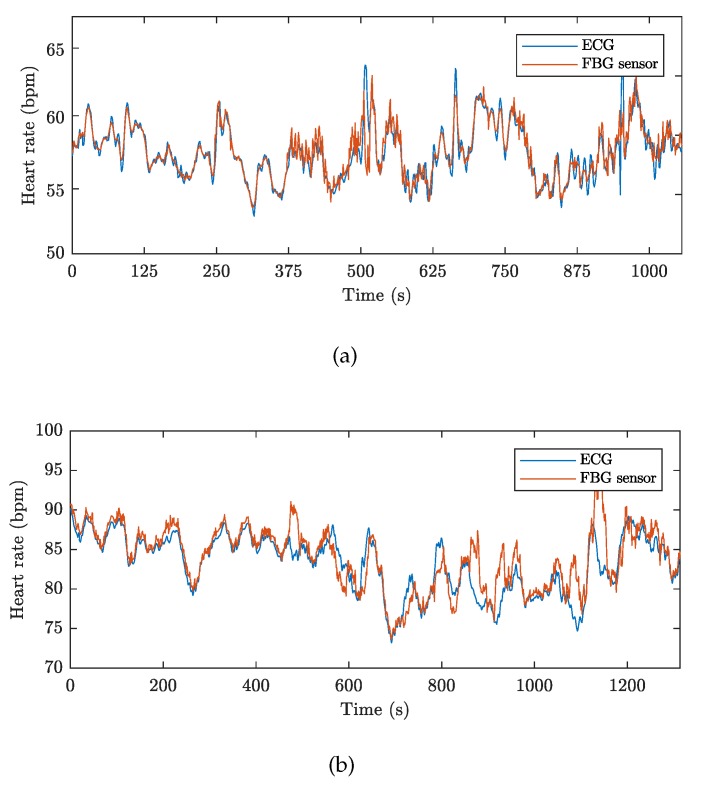
The full-time course of cardiac activity over the conducted MRI examinations: (**a**) volunteer M1, and (**b**) volunteer F1.

For better clarity we show graphs representing the development of the cardiac activity during the conducted MRI examinations in volunteers M1 and F1. Figure 14 represents a full-time course of cardiac activity (FBG sensor and reference) obtained from these test volunteers: M1 (17 min and 40 s) and F1 (21 min and 16 s). The HR is expressed in BPM (Beat Per Minute). The graphs show that the signal obtained from the FBG sensor quite accurately reproduces the signal obtained from the reference device. This fact (including the statistical data from the remaining 9 test volunteers) is described in Table 2 and is further confirmed by the Bland–Altman analysis.

**Figure 15:**
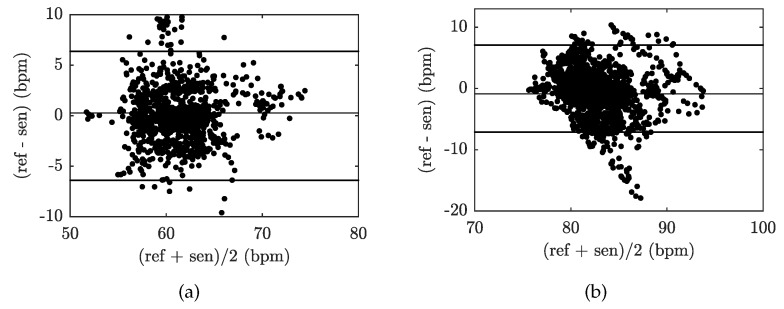
Reproducibility of HR determination capabilities of our FBG sensor (based on comparison with ECG-based HR calculations) using the Bland–Altman analysis method and data acquired from: (**a**) volunteer M1, and (**b**) volunteer F1.

Figure 15 shows the Bland–Altman graphs for the data acquired from the above-mentioned tested volunteers M1 and F1. The differences between the sensor and the reference traces, $x_{1}-x_{2}$, are plotted against their average values: $(x_{1}+x_{2})/2$.

A summary of the respiratory and heart rate measurements is provided in Table 2 and Table 3. The gender of the tested subjects is displayed as M1 to M6 for the males and F1 to F4 for the females. The Average Respiratory Rate (ARR) is expressed in Respiration Per Minute (RPM), the Average Heart Rate (AHR) is expressed in Beats Per Minute (BPM). The given values represent the ARR and AHR for the volunteers throughout the measurements. These tables also include the total recording time (Rec. time) for which the test volunteers were analyzed, Error represents the number of samples that were outside the ± 1.96 SD range, Relative error (Rel. error) indicates the number of erroneous samples relative to the total number of samples, expressed as a percentage. Further the total number of samples recorded by the FBG sensor (NoS sensor) and the results of the Bland–Altman analysis (Samples that were within ± 1.96 SD) are included in these tables. For RR measurements dataset, 95.36% of the values were within the ± 1.96 SD range. In the case of the HR measurements, 95.13% of the values were within the ± 1.96 SD range. The results obtained with the Bland–Altman analysis demonstrated and validated the functionality of the FBG sensor.

## 5. Discussion

Our FBG sensor can be conveniently placed in a clamping elastic belt. This belt can also be used in MR environments unless a respiratory reference is necessary. We need to emphasize that this sensor, with a relative error below 5% (±0.8 rpm for RR measurements and ±3.6 bmp for HR measurements), is designed primarily for monitoring rather than diagnosis.

**Figure 16:**
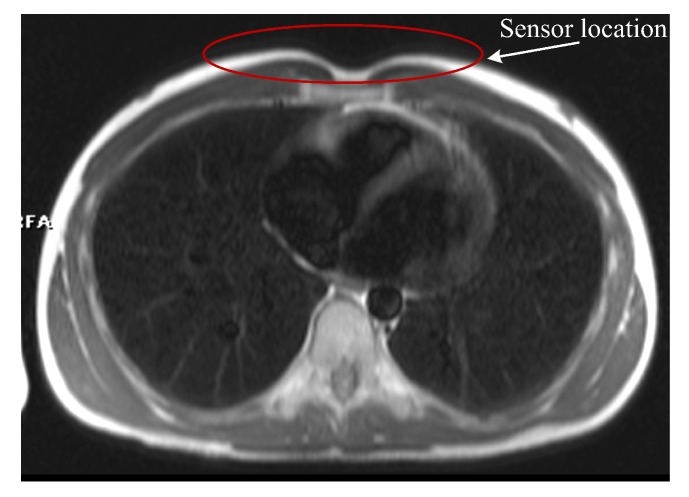
A sample MRI image (axial view) showing where the FBG sensor was positioned: A GE T2-weighted image (400 × 400 pixels).

During MRI examinations, the presence of the FBG sensor did not pose any safety issues to the test subjects and had no effect on the quality of the MR images. This fact is confirmed by Figure 16, which is an image of the region of the body over which the FBG sensor was placed. The FBG sensor is transparent to the MRI system and based on the acquired results we were able to verify that the FBG sensor does not introduce any artifacts into the spin-echo (SE) T1-weighted or to the gradient-echo (GE) T2-weighted imaging sequences.

**Figure 17:**
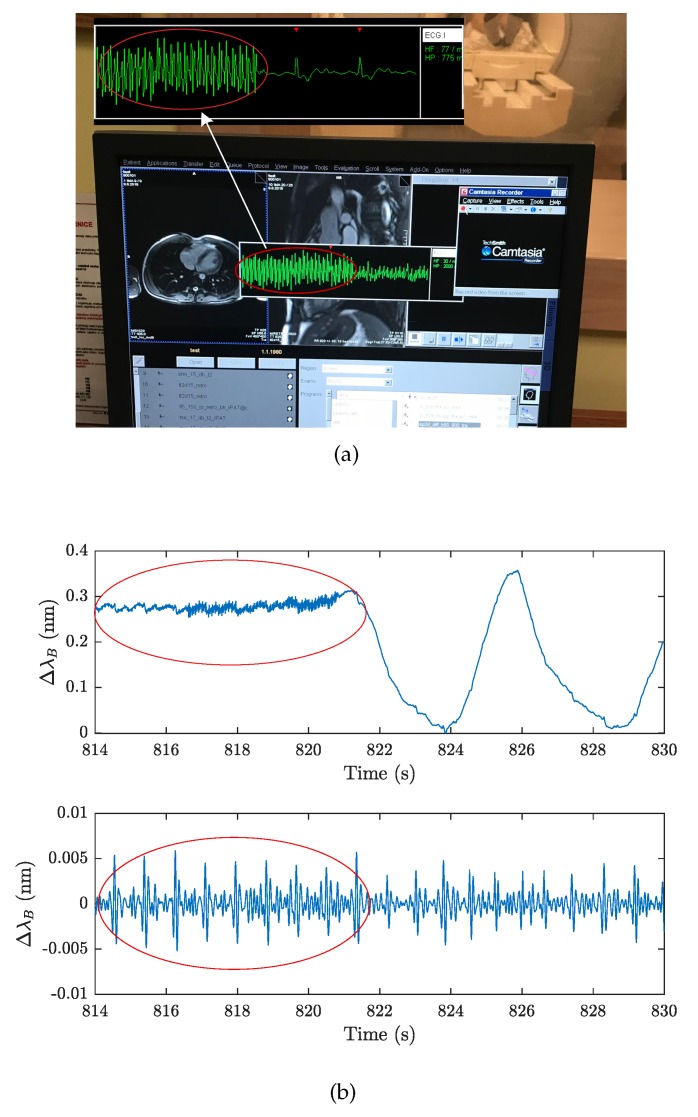
(**a**) An example of ECG signal interference in DWI MRI sequences, (**b**) An example of a signal obtained from the FBG sensor before and after filtering.

The problem with current ECG-based measurements during Diffusion Weighted Imaging is that the ECG signals may be degraded and the QRS complexes, or the R waves, would be difficult to detect. Currently this is a challenging research topic as detailed in [71,72]. Figure 17a shows a screen shot of the front panel of the MRI scanner used during our experimental measurements. In a more detailed view (encircled in red color), it is clear that the ECG signals are degraded by the DWI method to such an extent that the QRS complexes, or the R waves, cannot be clearly discerned. This results in a time lag from the start of the given sequences in each measurement cycle (this in turn extends the duration of the examination and leads into economic leakage due to the expensive MRI operation). As can be seen in Figure 17b, the critical time marks (encircled in red color) for the ECG signal were also recorded by the FBG sensor. However, by using the filtering operation described above, it is apparent that the J wave can be determined and detected. The sensor could therefore, serve as an alternative substitute for determining the J wave, which, as described in Section 3.3, corresponds to the R waves in these critical sequences that disturb the ECG signal. To confirm the above assumptions, we realize that there is a need for extensive research and, therefore, we are planning on undertaking this task in the near future.

**Figure 18:**
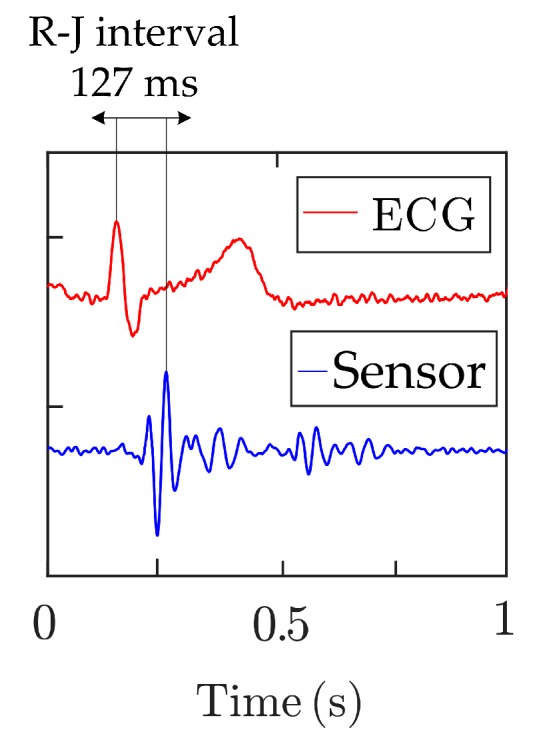
Example of analyzed R–J interval.

Within the measured group of ten healthy volunteers, we analyzed the time span of the R–J ranged from 121–143 ms. Figure 18 shows an example analyzed on a male volunteer. The measured values correspond to the results of the study [65] which is one of a few studies focused on the ballistocardiography signals—i.e., determine R–J interval.

In the first phase of our study, we focused on testing the basic functionality of the sensor. In the follow-up research based on the presented satisfactory results and information about time delay within the R–J interval, we focus on using the sensor for cardiac triggering at 1.5 T and 3 T (for synchronization and gating of cardiovascular magnetic resonance). To connect to the MR scanner, we will use the external input, but now we are working on the opto-electrical block, which will process, calculated and then streaming information of heart signals through the external input to the MR scanner.

In this article, we present an alternative method of non-invasive and cost-effective (the price of our sensor is approximately 100 dollars) MRI-compatible basic vital sign monitoring which has not been comprehensively explored yet. Despite the satisfactory results validated by the Bland–Altman’s analysis, we still find it necessary to perform clinical trials and strive towards achieving further technical improvements in our sensor design and fabrication. These steps would enable us to ascertain that our sensor could offer a reliable means to help clinicians for the cardiac triggering during MRI examinations or accurately detect the occurrence of hyperventilation states during MRI examinations and thereby prevent discomfort and complications that may arise for patients during such diagnostic procedures. We are positioning ourselves to carry out a comprehensive and long-term analysis of the functionality of this sensor in our follow-up research.

MR examination is based on the fact, that patients remain in the rest position (minimum body movement). On this fact is based the whole principle of measurement (ballistocardiography) described in this article by FBG sensor, because we detect (sense) the movements of the chest (respiratory rate) and the mechanical action of the heart (heart rate), but nevertheless, that patients can carry out slight body movements (minor and major artifacts) like a slight movement or trembling of the legs, torso rotation, slight hand movement, head movement, rapid breathing or coughing. If the above-mentioned minor/major artifact occurs, the signal may be negatively affected and the error rate in the evaluation may be increased (we must emphasize all of the different artifacts during measurements are taken into account in the results described above regarding the efficiency of the sensor). As for individual artifacts, some of the minor artifacts (shallow breathing, hand movement or head movement) did not distort the RR and HR measurements, but some major artifacts like a movement or rotation of torso caused degradation of the signal. In the follow-up research, we are ready to carry out a detailed analysis of the influence of the minor/major artifacts, but we must emphasize that this requires long-time extensive independent research. Every movement of the patient is individual and it is difficult to ensure the reproducibility of the research.

Compared to the results achieved by another FBG MR-similar studies (where statistics [73] were performed on 12 subjects with a recording length of only 5 min per person (total 60 min) or a similar study [38] on three subjects with a total recording length of 95 min or a similar study [59] on three subjects), we hope we presented statistically strong results (a total of 221 min of experimental data obtained by ten volunteers).

The sensor has been tested in the most conventionally used 1.5 T MR scanner and shows satisfactory results according to conventional medical devices. Since the sensor is based on fiber-optic technology and fiberglass material, which is immune to EMI, it can be assumed that the sensor can be also used within 3 T and 7 T MR scanners. Now, we are preparing measurements on a 3-T MR scanner with the cooperation of CEITEC MU (Brno, Czech Republic).

## 6. Conclusions

In this article, we described an innovative type of encapsulation for an FBG sensor suitable for basic vital sign monitoring (RR and HR determination). Sensor advantages against previously published works include a small size (30 × 10 × 0.8 mm) and light weight (2 g). The sensor is formed by a fiber-optic Bragg grating and fiberglass, which is a composite structure made up of glass fiber (fabric) and cured synthetic resin. Thanks to these desirable combinations, immunity to electromagnetic fields emanating from a variety of sources including supply networks, EMF generating devices, and others, is a primary characteristic feature of our FBG sensor. This characteristic allows monitoring the basic vital signs of the human body in the magnetic resonance, CT (computed tomography), X-ray, and other dense electromagnetic environments. The functionality of the sensor was verified in real 1.5 T MRI examinations on ten volunteers (both males and females) after acquiring their written informed consents. The Bland–Altman analysis showed 95.36% agreement between the measured RR data and the reference values, and 95.13% agreement between the HR measurements and reference values. These results clearly validated the functionality of our FBG sensor. The accuracy of this sensor was further characterized by a relative error below 5% (4.64% for RR and 4.87% for HR measurements).

The sensing technique described in this article can be successfully implemented to monitor respiratory and heart rates during 1.5 T MRI examinations. The results also confirmed the possibility of using the sensor for cardiac triggering at 1.5 T (for synchronization and gating of cardiovascular magnetic resonance) and for cardiac triggering when a Diffusion Weighted Imaging (DWI) is used.

## Figures and Tables

**Table 1:** A comparison of the most interesting prior works about fiber Bragg grating sensors tested in the real MR environments.

Reference	Sensor Size and Weight	Encapsulation Material	Quantitative Data on Sensor Efficiency
73	board 220 × 95 mm${}^{2}$; thickness 1.5 mm; weight: No data presented	polymethyl methacrylate (PMMA)	Twelve test subjects: maximum errors of RR and HR was within 7% (±1.2 rpm) and 6.5%(±3 bpm) of the average values
38	board 220 × 95 × 1.5 mm; weight: No data presented	polymethyl methacrylate (PMMA/Plexiglass)	Three test subjects: maximum relative errors of RR and HR was below 8% (7.67% and 6.61%)
59	PMMA board with dimmensions of 370 mm × 500 mm and thickness 3 mm; board 220 × 95 × 1.5 mm; weight: No data presented	(PMMA/Plexiglass)	Three test subjects: fractional error of determining HR was well below 7% (sitting and supine positions); maximum error was not more than 7.5% (standing position)
44	60 × 30 × 3 mm; weight: No data presented	Polydimethylsiloxane (PDMS)	Four test subjects: No data presented
57	50 × 50 × 5 mm; weight: No data presented	Polydimethylsiloxane (PDMS)	Four test subjects: maximum relative errors of RR and HR was 4.86% and 4.41%

**Table 2:** Summary of respiratory rate measurements.

Respiratory Rate						
Subject	Time (s)	ARR (rpm)	NoS	Error	Rel. Error	Samples in ±1.96 SD (%)
$M_{1}$	1041	14	272	11	4.04	95.96
$M_{2}$	1126	15	281	12	4.27	95.73
$M_{3}$	1312	17	372	15	4.03	95.97
$M_{4}$	1085	18	324	12	3.70	96.30
$M_{5}$	1419	15	356	14	3.93	96.07
$M_{6}$	1358	14	317	15	4.73	95.27
$F_{1}$	1276	16	307	18	5.86	94.14
$F_{2}$	1412	18	425	21	4.94	95.06
$F_{3}$	1095	16	296	17	5.74	94.26
$F_{4}$	1183	17	332	17	5.12	94.88
Sum	12,307		3282	152	4.64	95.36

**Table 3:** Summary of heart rate measurements.

Heart Rate						
Subject	Time (s)	AHR (bpm)	NoS	Error	Rel. Error	Samples in ±1.96 SD (%)
$M_{1}$	1041	61	1045	54	5.17	94.83
$M_{2}$	1126	68	1281	57	4.45	95.55
$M_{3}$	1312	74	1614	69	4.28	95.72
$M_{4}$	1085	65	1179	54	4.58	95.42
$M_{5}$	1419	71	1683	74	4.40	95.60
$M_{6}$	1358	70	1588	76	4.79	95.21
$F_{1}$	1276	84	1771	97	5.48	94.52
$F_{2}$	1412	82	1925	98	5.09	94.91
$F_{3}$	1095	86	1568	76	4.85	95.15
$F_{4}$	1183	82	1621	92	5.68	94.32
Sum	12,307		15,275	747	4.87	95.13

## References

[1] Weckesser M., Posse S., Olthoff U., Kemna L., Dager S., Müller-Gärtner H.W. (1999). Functional imaging of the visual cortex with bold-contrast MRI: Hyperventilation decreases signal response. Mag. Res. Med..

[2] Giardino N.D., Friedman S.D., Dager S.R. (2007). Anxiety, respiration, and cerebral blood flow: Implications for functional brain imaging. Compr. Psychiatry.

[3] Tamaki S., Yamada T., Okuyama Y., Morita T., Sanada S., Tsukamoto Y. (2009). Cardiac iodine-123 metaiodobenzylguanidine imaging predicts sudden cardiac death independently of left ventricular ejection fraction in patients with chronic heart failure and left ventricular systolic dysfunction: results from a comparative study with signal-averaged electrocardiogram, heart rate variability, and QT dispersion. J. Am. Coll. Cardiol..

[4] Zabel M., Acar B., Klingenheben T., Franz M.R., Hohnloser S.H., Malik M. (2000). Analysis of 12-lead T-wave morphology for risk stratification after myocardial infarction. Circulation.

[5] Ogura R., Hiasa Y., Takahashi T., Yamaguchi K., Fujiwara K., Ohara Y., Hosokawa S. (2003). Specific findings of the standard 12-lead ECG in patients with takotsubo’ cardiomyopathy. Circ. J..

[6] Chakeres D.W., Kanarlu A., Boudoulas H., Young D.C. (2003). Effect of static magnetic field exposure of up to 8 Tesla on sequential human vital sign measurements. J. Magn. Reson. Imaging.

[7] Jekic M., Ding Y., Dzwonczyk R. (2010). Magnetic field threshold for accurate electrocardiography in the MRI environment. Magn. Reson. Med..

[8] Krug J.W., Rose G. Magnetohydrodynamic distortions of the ECG in different MR scanner configurations. Proceedings of the 2011 Computing in Cardiology.

[9] Lanzer P., Barta C., Botvinick E.H., Wiesendanger H.U., Modin G., Higgins C.B. (1985). ECG-synchronized cardiac MR imaging: Method and evaluation. Radiology.

[10] Weissler A.M., Harris W.S., Schoenfeld C.D. (1968). Systolic Time Intervals in Heart Failure in Man. Circulation.

[11] Berne R.M., Levy M.N. (1997). Cardiovascular Physiology.

[12] Brau A.C., Wheeler C.T., Hedlund L.W., Johnson G.A. (2002). Fiber-optic stethoscope: A cardiac monitoring and gating system for magnetic resonance microscopy. Magn. Reson. Med..

[13] Baliyan V., Das C.J., Sharma R., Gupta A.K. (2016). Diffusion weighted imaging: Technique and applications. World J. Radiol..

[14] Biel L., Pettersson O., Philipson L., Wide P. (2001). ECG analysis: A new approach in human identification. IEEE Trans. Ind. Meas..

[15] Zhang Y., Hou Z. An algorithm for evaluating the ECG signal quality in 12 lead ECG monitoring system. Proceedings of the IEEE International Conference on Software Engineering and Service Sciences (ICSESS).

[16] Bousseljot R., Kreiseler D. (1998). ECG signal analysis by pattern comparison. Comput. Cardiol..

[17] Araoye M.A., Omotoso A.B., Opadijo G.O. (1998). The orthogonal and 12 lead ECG in adult negroes with systemic hypertension: comparison with age-matched control. West Afr. J. med..

[18] Tse Z.T.H., Dumoulin C.L., Clifford G.D., Schweitzer J., Qin L., Oster J., Jerosch-Herold M., Kwong R.Y., Michaud G., Stevenson W.G. (2014). 1.5 Tesla MRI-Conditional 12-lead ECG for MR Imaging and Intra-MR Intervention. Mag. Reson. Med..

[19] Dabaghyan M., Zhang S.H., Ward J., Kwong R.Y., Stevenson W.G., Watkins R.D., Zion T.T., Schmidt E.J. (2016). 3 T cardiac imaging with on-line 12-lead ECG monitoring. J. Cardiovasc. Mag. Reson..

[20] Frauenrath T., Hezel F., Renz W., D’Orth T., Dieringer M., Von Knobelsdorff-Brenkenhoff F., Prothmann M., Schulz-Menger J., Niendorf T. (2010). Acoustic cardiac triggering: A practical solution for synchronization and gating of cardiovascular magnetic resonance at 7 Tesla. J. Cardiovasc. Mag. Reson..

[21] Maderwald S., Orzada S., Lin Z., Schäfer L.C., Bitz A.K., Kraff O., Brote I., Häring L., Czylwik A., Zenge M.O. (2011). 7 Tesla Cardiac Imaging with a Phonocardiogram Trigger Device. Proc. Int. Soc. Mag. Reson. Med..

[22] Frauenrath T., Hezel F., Heinrichs U., Kozerke S., Utting J.F., Kob M., Butenweg C., Boesiger P., Niendorf T. (2009). Feasibility of cardiac gating free of interference with electro-magnetic fields at 1.5 Tesla, 3.0 Tesla and 7.0 Tesla using an MR-stethoscope. Investig. Radiol..

[23] Rotariu C., Cristea C., Arotaritei D., Bozomitu R.G., Pasarica A. Continuous respiratory monitoring device for detection of sleep apnea episodes. Proceedings of the 2016 IEEE 22nd International Symposium for Design and Technology in Electronic Packaging (SIITME).

[24] Vasanawala S.S., Jackson E. (2010). A method of rapid robust respiratory synchronization for MRI. Pediatr. Radiol..

[25] Yoon J.-W., Noh Y.-S., Kwon Y.-S., Kim W.-K., Yoon H.-R. (2014). Improvement of dynamic respiration monitoring through sensor fusion of accelerometer and gyro-sensor. J. Electr. Eng. Technol..

[26] Favero F.C., Villatoro J., Pruneri V. (2012). Microstructured optical fiber interferometric breathing sensor. J. Biomed. Opt..

[27] Sprager S., Donlagic D., Zazula D. Monitoring of basic human vital functions using optical interferometer. Proceedings of the IEEE 10th International Conference on Signal Processing (ICSP).

[28] Sprager S., Donlagic D., Zazula D. Estimation of heart rate, respiratory rate and motion by using optical interferometer as body sensor. Proceedings of the IASTED International Conference on Signal and Image Processing.

[29] Sprager S., Zazula D. (2013). Detection of heartbeat and respiration from optical interferometric signal by using wavelet transform. Comput. Methods Prog. Biomed..

[30] Will C., Shi K., Lurz F., Weigel R., Koelpin A. Intelligent signal processing routine for instantaneous heart rate detection using a Six-Port microwave interferometer. Proceedings of the 2015 International Symposium on Intelligent Signal Processing and Communication Systems (ISPACS).

[31] Zazula D., Sprager S. Detection of the first heart sound using fibre-optic interferometric measurements and neural networks. Proceedings of the Symposium on Neural Network Applications in Electrical Engineering.

[32] Byeong H.L., Young H.K., Kwan S.P., Joo B.E., Myoung J.K., Byung S.R., Hae Y.C. (2012). Interferometric Fiber Optic Sensors. Sensors.

[33] Hsieh Y.H., Chen N.K. Micro tapered Mach–Zehnder fiber interferometer for monitoring pressure fluctuation and its applications in pulse rate detection. Proceedings of the 2013 6th IEEE/International Conference on Advanced Infocomm Technology (ICAIT).

[34] Roriz P., Carvalho L., Frazão O., Santos J.L., Simoes A.J. (2014). From conventional sensors to fibre optic sensors for strain and force measurements in biomechanics applications: A review. J. Biomech..

[35] Dziuda L. (2015). Fiber-optic sensors for monitoring patient physiological parameters: A review of applicable technologies and relevance to use during magnetic resonance imaging procedures. J. Biomech..

[36] Chethana K., Guru Prasad A.S., Omkar S.N., Asokan S. (2016). Fiber bragg grating sensor-based device for simultaneous measurement of respiratory and cardiac activities. J. Biophotonics.

[37] Dzuida L., Skibniewski F.W., Krej M., Lewandowski J. (2012). Monitoring respiration and cardiac activity using fiber Bragg grating-based sensor. IEEE Trans. Biomed. Eng..

[38] Dziuda L., Krej M., Skibniewski F.W. (2013). Fiber Bragg grating strain sensor incorporated to monitor patient vital signs during MRI. IEEE Sens. J..

[39] Emamian M., Hasanian S.M., Tayefi M., Bijari M., Movahedian far F., Shafiee M., Avan A., Heidari-Bakavoli A., Moohebati M., Ebrahimi M. (2017). Association of hematocrit with blood pressure and hypertension. J. Clin. Lab. Anal..

[40] Hang-yin L. (2005). Experimental study of non-uniform strains in composites with embedded fiber bragg grating. Meas. Sci. Technol..

[41] Anoshkin A.N., Shipunov G.S., Voronkov A.A., Shardakov I.N. (1909). Effect of temperature on the spectrum of fiber Bragg grating sensors embedded in polymer composite. AIP Conf. Proc..

[42] Zhang Y. (2018). The Packaging Technology Study on Smart Composite Structure Based on the Embedded FBG Sensor. IOP Conf. Ser. Mater. Sci. Eng..

[43] Fajkus M., Nedoma J., Martinek R., Vasinek V., Nazeran H., Siska P. (2017). A Non-invasive Multichannel Hybrid Fiber-optic Sensor System for Vital Sign Monitoring. Sensors.

[44] Nedoma J., Kepak S., Fajkus M., Cubik J., Siska P., Martinek R., Krupa P. (2018). Magnetic Resonance Imaging Compatible Non-Invasive Fibre-Optic Sensors Based on the Bragg Gratings and Interferometers in the Application of Monitoring Heart and Respiration Rate of the Human Body: A Comparative Study. Sensors.

[45] Yang X., Chen Z., Elvin C.S.M., Janice L.H.Y., Ng S.H., Teo J.T., Wu R. (2015). Textile Fiber Optic Microbend Sensor Used for Heartbeat and Respiration Monitoring. IEEE Sens. J..

[46] Grilett A., Kinet D., Witt J., Schukar M., Krebber K., Pirotte F., Depre A. (2008). Optical fiber sensors embedded into medical textiles for healthcare monitoring. IEEE Sens. J..

[47] Chen D., Lau J.T., Teo S.H., Ng X., Yang P., Kei L. (2014). Simultaneous measurement of breathing rate and heart rate using a microbend multimode fiber optic sensor. J. Biomed. Opt..

[48] Krehel M., Schmid M., Rossi R.M., Boesel F.L., Bona G.L., Scherer L.J. (2014). An optical fibre-based sensor for respiratory monitoring. Sensors.

[49] Leal-Junior A.G., Díaz C.R., Leitão C., Pontes M.J., Marques C., Frizera A. (2018). Polymer optical fiber-based sensor for simultaneous measurement of breath and heart rate under dynamic movements. Opt. Laser Technol..

[50] Taffoni F., Formica D., Saccomandi P., Di Pino G., Schena E. (2013). Optical fiber-based MR-compatible sensors for medical applications: An overview. Sensors.

[51] Grillet A., Kinet D., Witt J., Schukar M., Krebber K., Pirotte F., Depré A. Optical fibre sensors embedded into medical textiles for monitoring of respiratory movements in MRI environment. Proceedings of the Third European Workshop on Optical Fibre Sensors.

[52] Moerman K.M., Sprengers A.M.J., Nederveen A.J., Simms C.K. (2013). A novel MRI compatible soft tissue indentor and fibre Bragg grating force sensor. Med. Eng. Phys..

[53] Tan U.-X., Yang B., Gullapalli R., Desai J.P. (2011). Triaxial MRI-compatible fiber-optic force sensor IEEE. Trans. Robot..

[54] Yoo W.J., Jang K.W., Seo J.K., Heo J.Y., Moon J.S., Park J.Y., Lee B. (2010). Development of respiration sensors using plastic optical fiber for respiratory monitoring inside MRI system. J. Opt. Soc. Korea.

[55] Kam W., Mohammed W.S., Leen G., O’Sullivan K., O’Keeffe M., O’Keeffe S., Lewis E. All plastic optical fiber-based respiration monitoring sensor. Proceedings of the IEEE Sensors.

[56] Filograno M.L., Pisco M., Catalano A., Forte E., Aiello M., Cavaliere C., Soricelli A., Davino D., Visone C., Cutolo A. (2017). Triaxial Fiber Optic Magnetic Field Sensor for Magnetic Resonance Imaging. J. Lightw. Technol..

[57] Nedoma J., Fajkus M., Novak M., Strbikova N., Vasinek V., Nazeran H., Vanus J., Perecar F., Martinek R. (2017). Validation of a novel fiber-optic sensor system for monitoring cardiorespiratory activities during mri examinations. Adv. Electr. Electron. Eng..

[58] Su H., Shang W., Li G., Patel N., Fischer G.S. (2017). An MRI-Guided Telesurgery System Using a Fabry-Perot Interferometry Force Sensor and a Pneumatic Haptic Device. Ann. Biomed. Eng..

[59] Dziuda L., Skibniewski F.W. (2014). A new approach to ballistocardiographic measurements using fibre Bragg grating-based sensors. Biocybern. Biomed. Eng..

[60] Agarwal R.P., O’Regan D. (2009). Two-Dimensional Wave Equation. Ordinary and Partial Differential Equations.

[61] Kersay A.D., Davis M.A., Patrick H.J., LeBlanc M., Koo K.P., Askins C.G., Putnam M.A., Friebele E.J. (1997). Fiber grating sensors. J. Lightw. Technol..

[62] Pinheiro E., Postolache O., Girao P. (2010). Theory and Developments in an Unobtrusive Cardiovascular System Representation: Ballistocardiography. Open Biomed. Eng. J..

[63] Lindqvist A., Pihlajamäki K., Jalonen J., Laaksonen V., Alihanka J. (1996). Static-charge-sensitive bed ballistocardiography in cardiovascular monitoring. Clin. Physiol..

[64] Inan O.T. (2011). Novel Technologies for Cardiovascular Monitoring Using Ballistocardiography and Electrocardiography.

[65] Wiard R.M., Inan O.T., Giovangrandi L., Cuttino C.M., Kovacs G.T.A. Preliminary results from standing ballistocardiography measurements in microgravity. Proceedings of the 2013 35th Annual International Conference of the IEEE Engineering in Medicine and Biology Society (EMBC).

[66] Zhu Y., Zhang H., Jayachandran M., Ng A.K., Biswas J., Chen Z. Ballistocardiography with fiber optic sensor in headrest position: A feasibility study and a new processing algorithm. Proceedings of the 2013 35th Annual International Conference of the IEEE Engineering in Medicine and Biology Society (EMBC).

[67] Jiao C., Lyons P., Zare A., Rosales L., Skubic M. Heart beat characterization from ballistocardiogram signals using extended functions of multiple instances. Proceedings of the Annual International Conference of the IEEE Engineering in Medicine and Biology Society EMBS.

[68] Product FBGuard. http://www.safibra.cz/en/fbguard-interrogation-unit.

[69] Product TSD221-MRI. https://www.biopac.com/wp-content/uploads/TSD221-MRI.pdf.

[70] Bland J.M., Altman D.G. (1999). Measuring agreement in method comparison studies. Stat. Methods Med. Res..

[71] Yang L., Xu L., Schoepf U.J., Wichmann J.L., Fox M.A., Yan J., Fan Z., Zhang Z. (2015). Prospectively ECG-triggered sequential dual-source coronary CT angiography in patients with atrial fibrillation: Influence of heart rate on image quality and evaluation of diagnostic accuracy. PLoS ONE.

[72] Stäb D., Roessler J., O’Brien K., Hamilton-Craig C., Barth M. (2016). ECG Triggering in Ultra-High Field Cardiovascular MRI. Tomography.

[73] Dziuda L., Skibniewski F.W., Krej M., Baran P.M. (2013). Fiber Bragg grating-based sensor for monitoring respiration and heart activity during magnetic resonance imaging examinations. J. Biomed. Opt..

